# Implementing core NICE guidelines for osteoarthritis in primary care with a model consultation (MOSAICS): a cluster randomised controlled trial

**DOI:** 10.1016/j.joca.2017.09.010

**Published:** 2018-01

**Authors:** K.S. Dziedzic, E.L. Healey, M. Porcheret, E.K. Afolabi, M. Lewis, A. Morden, C. Jinks, G.A. McHugh, S. Ryan, A. Finney, C. Main, J.J. Edwards, Z. Paskins, A. Pushpa-Rajah, E.M. Hay

**Affiliations:** † Arthritis Research UK Primary Care Centre, Research Institute for Primary Care & Health Sciences, Keele University, Keele, Staffordshire, ST5 5BG, UK; ‡ School of Social and Community Medicine, University of Bristol, Gloucestershire, UK; § School of Healthcare, University of Leeds, LS2 9JT, UK; ‖ The Haywood Hospital, High Lane, Burslem, Stoke-on-Trent Staffordshire, ST6 7AG, UK; ¶ Dermatology, King's College London, Tower Wing Guy's, London, WC2R 2LS, UK

**Keywords:** Osteoarthritis, Primary care, Implementation, NICE guidelines, Self-management, Quality indicators

## Abstract

**Objective:**

To determine the effectiveness of a model osteoarthritis consultation, compared with usual care, on physical function and uptake of National Institute for Health and Care Excellence (NICE) osteoarthritis recommendations, in adults ≥45 years consulting with peripheral joint pain in UK general practice.

**Method:**

Two-arm cluster-randomised controlled trial with baseline health survey. Eight general practices in England. Participants: 525 adults ≥45 years consulting for peripheral joint pain, amongst 28,443 population survey recipients. Four intervention practices delivered the model osteoarthritis consultation to patients consulting with peripheral joint pain; four control practices continued usual care.

The primary clinical outcome of the trial was the SF-12 physical component score (PCS) at 6 months; the main secondary outcome was uptake of NICE core recommendations by 6 months, measured by osteoarthritis quality indicators. A Linear Mixed Model was used to analyse clinical outcome data (SF-12 PCS). Differences in quality indicator outcomes were assessed using logistic regression.

**Results:**

525 eligible participants were enrolled (mean age 67.3 years, SD 10.5; 59.6% female): 288 from intervention and 237 from control practices. There were no statistically significant differences in SF-12 PCS: mean difference at the 6-month primary endpoint was −0.37 (95% CI −2.32, 1.57). Uptake of core NICE recommendations by 6 months was statistically significantly higher in the intervention arm compared with control: e.g., increased written exercise information, 20.5% (7.9, 28.3).

**Conclusion:**

Whilst uptake of core NICE recommendations was increased, there was no evidence of benefit of this intervention, as delivered in this pragmatic randomised trial, on the primary outcome of physical functioning at 6 months.

**Trial registration:**

ISRCTN06984617.

## Introduction

Osteoarthritis (OA) is a major cause of pain and disability in older adults: musculoskeletal pain in adults aged 45 years and over is the number one cause of years lived with disability worldwide^1^. Routine OA management in UK general practice has been found to lack adherence to guidelines produced by the National Institute for Health and Care Excellence (NICE)^2^, updated in 2014^3^, especially for ‘core’ self-management approaches such as written information, exercise and weight loss^4^, ^5^, ^6^. Implementation of the NICE recommendations has not yet been evaluated in UK general practice.

Healthcare professionals often frame consultations in terms (such as ‘wear and tear’) thought to reassure patients or be patient-friendly which may have a negative impact^7^. Patients and general practitioners (GPs) want more advice and support on understanding OA and the use of non-pharmacological approaches^6^, ^7^. Patient perceived health service needs have also been found to align with clinical guideline recommendations^8^.

The MOSAICS (Managing OSteoArthritis In ConsultationS) study was a cluster-randomised controlled trial to investigate the effectiveness of a complex intervention – a model OA consultation (MOAC) – on clinical outcomes, and on the uptake of core NICE OA core recommendations in participants aged ≥45 years consulting their GP with peripheral joint pain (hand, hip, knee, foot).

## Methods

### Design

The MOSAICS study had two key parts: (1) a population health survey that took place between May 2011 and April 2012, prior to (2) a two-arm cluster-randomised controlled trial conducted in eight general practices in Cheshire, Shropshire or Staffordshire, UK. The protocol has been published^9^ and we have previously reported the practice-level evaluation of the intervention using anonymised medical records^10^. By using medical record information for measuring the outcomes, all eligible patients in the practices were included but no patient reported outcomes were analysed by Jordan *et al.*^10^. Here we report the patient-level evaluation of the clinical effectiveness of the MOAC intervention in patients with OA and describe the uptake of core NICE OA recommendations for those patients who gave consent to be part of the clinical outcomes study.

Given the practice-level unit of randomisation, it was important to avoid the potential for bias in selection and recruitment of participants. We used a population health survey to pre-determine potentially eligible participants prior to consultation for joint pain to establish baseline characteristics as the majority of the population are registered with a GP in England. This was mailed to all patients aged ≥45 years eligible to receive a postal survey and registered with one of the eight general practices participating in the MOSAICS study. Survey participants were asked questions about any joint pain and general health, as well as for permission for further contact and medical record review. Those who subsequently consulted their GP for joint pain during the trial recruitment phase were invited to take part in the cluster trial. Eligibility of potential participants for the cluster trial was identified at this stage and GPs and practice nurses therefore played no role in determining eligibility for recruitment to the cluster trial.

The cluster trial was conducted from May 2012 through to February 2014 by the Arthritis Research UK Primary Care Centre, Keele University, UK.

### Setting and participants

#### General practices

Ten general practices, all using the EMIS electronic health records (EHR) system, were invited to participate. Eight practices consented to take part. Eligibility of practices has been described elsewhere^9^. Reasons for non-participation included recent engagement with teaching medical students and other research involvement. The combined population of patients aged ≥45 years registered with the eight participating general practices (estimate 30,000) formed the study sampling frame. Resources to support general practice engagement were offered via the UK National Institute of Health Research Clinical Research Network^9^.

During a 6 month run-in period, all practices received a resource pack of written advice with examples of patient leaflets about OA provided by Arthritis Research UK and Arthritis Care. An OA consultation e-template was designed to collect information on quality indicators of OA care^11^. The e-template was installed in all eight practices for the 6 month baseline period prior to randomisation, to make the recording of joint pain consultations part of routine care and determine any effect of the e-template on practice. The e-template was triggered in consultations through entry of any Read system morbidity code for clinical OA (peripheral joint pain – hand, hip, knee, foot); these same Read codes were used to identify patients for the trial. The effects of the e-template have previously been reported^11^. Briefly, the e-template was associated with increased recording of weight measurement and increased prescription of NICE-recommended analgesics (topical NSAIDs, paracetamol) in the run-in period, but other care remained stable.

Following the 6-month run in period, practices were randomised into intervention (four practices) or usual care (four practices). All eight practices continued to use the e-template introduced at baseline^9^, ^11^ to routinely record care in all consultations for joint pain during the study period regardless of subsequent recruitment to the trial.

Eligibility criteria for the health survey and the trial are described in Dziedzic *et al*.^9^ and Appendix 1.

#### Participants

Eligible registered adults (Dziedzic *et al*.^9^, and Appendix 1) from the eight practices aged ≥45 years were mailed a health survey between May 2011 and February 2012. Potential trial participants were survey responders reporting peripheral joint pain who provided written consent to further contact and medical record review. Those who subsequently consulted their GP for peripheral joint pain during the 9 month recruitment phase (from April to December 2012) were invited to take part in the cluster trial. Fortnightly searches in the medical records identified when the OA template had been opened on tagged records which allowed for identification of eligible participants.

Invitations were mailed 2 weeks after the GP consultation, together with a study information sheet and a questionnaire (the ‘post-consultation baseline’) on joint pain, self-management approaches, health status and resource use^9^.

#### Randomisation

Following the six-month run-in period, general practices were randomly allocated by administrative staff at the Keele Clinical Trials Unit (who had no clinical involvement in the trial) to two arms using a computer random number generator with block randomisation stratified by practice list size (block size, 4): to intervention (MOAC) plus e-template (*n* = 4) or control (usual care) plus e-template (*n* = 4). The Principal Investigator and trial administrative members who entered the data were unaware of allocation. The trial statisticians were kept blind to the allocation until after the intention-to-treat analysis (blinding was broken for per-protocol analysis).

#### Intervention

Practices delivered the MOAC described in full in Appendix 2, which consisted of: an enhanced GP consultation to make, give and explain the diagnosis, and provide initial care for older adults presenting with peripheral joint pain; an OA Guidebook offered by the GP to patients to support OA self-management (https://www.keele.ac.uk/media/keeleuniversity/ri/primarycare/pdfs/OA_Guidebook.pdf); advice on analgesia; and up to four follow-up practice nurse consultations to guide patients in self-management for OA with advice on weight management if required, general exercise, and physical activity, with goal-setting as appropriate. The development of the intervention has been published elsewhere^9^, ^12^, ^13^. Briefly, the intervention followed the Whole Systems Informing Self-Management Engagement (WISE) model for guided self-management^14^ including provision of patient information (the OA guidebook)^13^, care responsive to patient needs^15^, and good access to follow-up care (practice nurse consultations). Appendix 2 also provides full details of the training for GPs and practice nurses.

#### Control

Control practices received no training, guidebook or dedicated nurse OA clinic and continued usual care as in the pre-randomisation period.

#### Patient-level evaluation

The primary outcome for clinical effectiveness was the SF-12 physical component score (PCS) at 6 months^16^. Uptake of NICE core recommendations during the 6 months following the index consultation was measured by self-reported quality indicators of OA care^17^. Self-management and patient enablement were also measured by questionnaires^9^, ^18^.

Secondary outcomes included measures of pain (peripheral joint pain intensity, OMERACT/OARSI responder criteria^19^), joint problem self-management (Arthritis Self- Efficacy pain subscale), physical activity (IPAQ, Physical Activity Scale for the Elderly [PASE]), and Global Assessment of Change^9^. For further details of OMERACT/OARSI responder criteria see footnote to Supplementary file, Table III. Measures of mental health included the SF-12 mental component summary (MCS), the eight-item Patient Health Questionnaire depression scale (PHQ8) and seven-item Generalised Anxiety Disorder Questionnaire (GAD7)^9^.

Questionnaires were administered by mail after the index consultation (‘post-consultation baseline’) and at three, six and 12 months to determine short, medium and longer term outcomes. Non-responders were invited to complete a minimum data collection. The EQ-5D outcome measure was collected to inform the cost-effectiveness analysis, to be reported separately.

### Treatment fidelity

To investigate the extent to which participants received the practice nurse component of the MOAC intervention, the content, number and percentage of participants in the intervention arm having had a practice nurse consultation for OA were identified from case report forms and medical records.

### Sample size

With no prior data on quality indicators of OA in UK primary care, we used the primary clinical outcome (SF-12 PCS) for the sample size calculation. In total, 500 participants were needed at baseline, allowing for a 20% drop-out, to detect the effect size of 0.3 (‘small to moderate’) with 90% power and 5% two-tailed significance at the primary time-point of 6 months^9^. The sample size calculation was adjusted to correct for: clustering (adjusted intracluster correlation coefficient (ICC) of 0.005); varying practice size recruitment (including coefficient of variation of 0.5); and repeated-measures design and dropout (×0.67 and  × 1.25 respectively)^9^.

### Analysis

Baseline trial characteristics were compared between treatment arms and presented at the level of: (1) trial arms, and (2) participant characteristics. Longitudinal linear mixed models were used to analyse health outcomes: a 3-level hierarchical analysis was carried out accounting for clustering at the levels of GP-Practice and individual participants through repeated measures across time 0 (baseline), 3, 6 and 12 months – including time × group interactions to estimate the treatment effect across the three follow up timepoints. Fixed-covariate adjustments were made for age, gender, baseline SF-12 PCS, corresponding patient baseline score and practice size (specified a priori within the analysis plan). All baseline responders were included in the dataset and the analyses accounted for missing data under the ‘missing at random’ (MAR) assumption by modelling the interaction of baseline covariates and time – hence retaining the intention-to-treat principle. For dichotomous ‘quality-indicators’ outcomes, multiple imputation was used to account for missing data (assuming MAR) with odds ratio estimates derived from 2-level hierarchical logistic regression models adjusted for age, gender and practice size (with GP-Practice as random factor): Absolute percent difference estimates were calculated through applying derived odds ratios (intervention vs control (reference)) to observed prevalence figures in the control arm. Statistical significance is at the 5% (two tailed) level. Analysis was carried using SPSS v.21 (IBM Corp, 2012) and STATA v.13.0/14.0 (Stata Corp, 2013/5). Sensitivity analyses were conducted for the primary clinical outcome (SF-12 PCS) (Detailed in Supplementary file, Table I).

## Results

### Study recruitment and follow-up

**Fig. 1:**
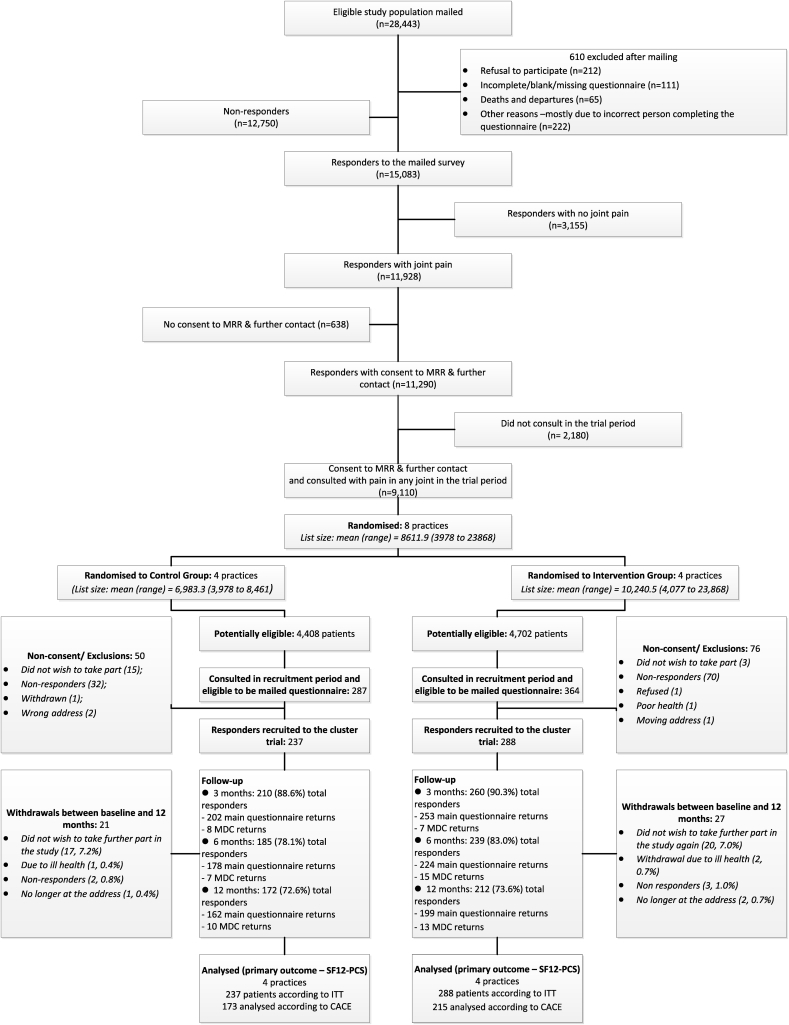
Study flow chart.

Mean (SD) practice size for the four intervention practices was 10,240.5 (9174.8) and mean number of GPs was 6.0 (6.1), compared with 6983.3 (2060.7) and 5.2 (2.9) respectively for the four control practices. Trial eligibility, recruitment and follow-up are shown in Fig. 1. Of 15,083 eligible responders reporting joint pain and consenting to medical record review in the health survey, 651 participants subsequently consulted for peripheral joint pain during the 6 month recruitment period and were invited to take part in the cluster trial. 525 consented with 288 patients recruited from intervention practices and 237 from control practices.

The median (inter-quartile range) time between the index consultation and response to the post-consultation baseline questionnaire was 28 (21,40) days for the intervention group, 29 (22,40) for the control group. The mean age (SD) was 67.3 years (10.5); 59.6%, were female; 81% had multisite pain (pain in two or more of hand, hip, knee, foot). Overall, differences in participant characteristics across treatment arms at post-consultation baseline were small (Table I). Overall follow-up rates (including minimum data collection) were: 3 months, *n* = 470 (89.5%), 6 months, *n* = 424 (80.8%), 12 months, *n* = 384 (73.1%). Rates of loss to follow-up were similar for both trial arms (Fig. 1).

**Table I:** Summary of General Practice (GP) and individual participant characteristics at baseline by study group

GP Practice characteristics∗	Intervention *n* = 4	Control *n* = 4
**Practice size**, mean (SD)	10,240 (9174.8)	6983 (2060.7)
**Practice Index of Multiple Deprivation rank**, median (IQR)	9165.0 (2195.7, 19,478.5)	14,633.5 (4571.5, 28,822.0)
**Number of General Practitioners**, mean (SD)	6.0 (6.1)	5.5 (2.9)
**Age (years) of GP**, mean (SD)	42.2 (23.7)	42.8 (23.5)
**Participants characteristics**	**Intervention n** = **288**	**Control n** = **237**
**Gender**, *n* (%)		
Female	167 (58.0)	146 (61.6)
Male	121 (42.0)	91 (38.4)
**Age** (years) mean (SD)	66.9 (10.6)	67.7 (10.3)
**BMI** (kg m^−2^), mean (SD)	28.1 (5.1)	28.5 (4.8)
**Marital status**, *n* (%)		
Married	186 (65.0)	168 (71.0)
Separated	2 (0.7)	4 (1.7)
Divorced	29 (10.1)	13 (15.6)
Widowed	44 (15.4)	37 (15.6)
Cohabiting	10 (3.5)	9 (3.8)
Single	15 (5.2)	6 (2.5)
**Employment status**, *n* (%)		
Employed	77 (27.2)	59 (25.2)
Not working/retired	206 (72.8)	175 (74.8)
**Deprivation index**		
Median (IQR)	21,868 (15,144, 28,649)	20,182 (15,989, 24,635)
**No. of pain sites**, *n* (%)		
1	55 (19.1)	45 (19.0)
2 or more	233 (80.9)	192 (81.0)

∗ Age and gender structure of the registered population at the practices was similar to that of North Staffordshire and of England and Wales. Practices had a range of numbers of patients, a range of areas – semi rural to urban (small town/larger city), and a range of deprivation.

### Clinical effectiveness

At 6 months difference between intervention and control arms for the primary clinical outcome (Table II) was not statistically significant (*P* ≥ 0.05) after adjustment for predefined potential confounders. Mean difference in the SF-12 PCS at 6 months (primary analysis) was −0.37 (95% CI: −2.32, 1.57) for intervention compared to the control group, which was neither clinically nor statistically significant; equating to a standardised mean difference (effect size relative to baseline SD of 11.26) of: −0.03 (95% CI: −0.21, 0.14). The crude (unadjusted) intracluster correlation (ICC) was small: 0.006 (less than 0.001 when adjusting for baseline).

**Table II:** Effectiveness of the model osteoarthritis consultation on the primary outcome measure (SF-12 PCS) compared to usual primary care for osteoarthritis

SF-12 PCS	Intervention			Control			∗Mean difference (95% CI)	†Effect size (95% CI)	*P*-value
	Valid *n*	Mean	SD	Valid *n*	Mean	SD			
Post-consultation	280	36.49	11.48	231	36.48	11.00	–	–	–
3 months	250	38.03	12.32	204	38.12	11.58	−0.29 (−1.86, 1.29)	−0.03 (−0.17, 0.11)	0.722
6 months	229	38.99	12.12	180	38.89	12.00	−0.37 (−2.32, 1.57)	−0.03 (−0.21, 0.14)	0.706
12 months	200	38.79	12.58	166	39.22	11.84	−0.90 (−3.75, 1.96)	−0.08 (−0.33, 0.17)	0.539

ICC: 0.006 (unadjusted); <0.001 (adjusted for baseline score).

∗ Calculated as mean difference for Intervention group – control group by linear mixed modelling adjusted for age, gender, practice size and baseline SF-12 PCS (clustering by general practice accounted for in the mixed model).

† Mean difference relative to pooled ‘baseline’ (post-consultation) SD.

### Uptake of self-management and NICE recommendations

Differences between intervention and control arms for the self-reported management offered during the 6 months after the index consultation were statistically significantly greater in the intervention arm compared with control for core NICE OA recommendations: information/advice about exercises (% difference [95% CI]) 20.5% (7.9%, 28.3%); and paracetamol for pain 10.7 (0.6%, 20.7%) (Table III).

**Table III:** Self-report quality indicators of osteoarthritis care and treatment used within the first 6 months of consultation

Self-reported OA quality indicators	Intervention	Control	OR (95% CI)	Absolute % difference^Δ^ (95% CI)	*P*-value
**Treatment offered**					
‡Education, advice and access to information	95.0%	91.5%	2.95 (0.68, 12.8)	5.4% (−3.5%, 7.7%)	0.148
Support on how to help self with joint problem	66.9%	60.1%	1.91 (0.95, 3.81)	14.1% (−1.1%, 25.1%)	0.068
Information/advice about exercises, muscle strengthening or physical activities	81.5%	63.3%	3.01 (1.43, 6.32)	20.5% (7.9%, 28.3%)	0.004
Referral to strengthening or physical activities	54.4%	46.9%	1.45 (0.85, 2.55)	9.2% (−3.9%, 22.3%)	0.126
#Advice to lose weight	46.3%	43.0%	1.33 (0.79, 2.24)	7.0% (−5.7%, 19.8%)	0.288
#Referral to services for losing weight	16.4%	12.9%	2.92 (0.85, 9.98)	17.3% (−1.7%, 46.7%)	0.087
Paracetamol recommended for pain	79.7%	70.3%	1.80 (1.03, 4.25)	10.7% (0.6%, 20.7%)	0.037
Stronger painkiller	69.4%	68.8%	1.18 (0.71, 1.95)	3.4% (−7.8%, 12.3%)	0.529
Information about drugs effect provided	68.2%	72.6%	0.65 (0.39, 1.09)	−9.2% (−21.6%, 1.6%)	0.101
Corticosteroid joint injection	35.2%	35.8%	1.12 (0.64, 1.84)	2.6% (−9.6%, 14.8%)	0.677
Surgery evaluation	32.3%	37.0%	0.82 (0.42, 1.62)	−4.4% (−17.3%, 11.8%)	0.574
Need for walking aid assessed	28.9%	28.9%	1.05 (0.60, 1.65)	1.0% (−9.3%, 11.3%)	0.853
Need for appliances/aids to daily living	14.4%	18.2%	0.91 (0.45, 1.82)	−1.4% (−9.1%, 10.6%)	0.780
**Treatment used**					
‡Education, advice and access to information	62.0%	47.6%	2.67 (1.62, 4.40)	23.2% (11.9%, 32.4%)	<0.001
Muscle strengthening exercises	60.5%	44.3%	1.91 (1.20, 3.20)	16.0% (4.4%, 27.5%)	0.007
General fitness exercises	38.0%	35.4%	0.80 (0.45, 1.29)	−4.8% (−15.6%, 6.0%)	0.384
Physiotherapy	40.1%	38.6%	0.65 (0.38, 1.13)	−9.5% (−19.4%, 2.9%)	0.126
#Dieting to lose weight	48.4%	50.9%	0.87 (0.52, 1.44)	−3.6% (−16.0%, 8.9%)	0.577
Paracetamol	86.5%	84.8%	1.24 (0.63, 2.44)	2.6% (−7.0%, 8.4%)	0.535
Anti-inflammatory creams/gels e.g., topical NSAIDs	81.6%	79.8%	1.21 (0.67, 2.21)	2.9% (−7.3%, 9.9%)	0.527
Capsaicin cream	21.8%	19.4%	1.55 (0.87, 2.77)	7.7% (−2.2%, 20.6%)	0.141
Anti-inflammatory tablets, e.g., oral NSAIDs	59.9%	70.6%	0.51 (0.31, 0.85)	−15.6% (−28.3%, −3.5%)	0.010
Stronger painkillers, e.g., Opioids	62.9%	62.6%	1.12 (0.70, 1.80)	2.7% (−8.6%, 12.5%)	0.626
Community pharmacy	25.8%	16.5%	1.84 (1.02, 3.34)	10.2% (0.3%, 23.3%)	0.043
Walking aids	41.9%	50.4%	0.57 (0.34, 0.94)	−13.9% (−24.6%, −1.6%)	0.027
Shock-absorbing shoes or insoles	34.8%	31.8%	1.34 (0.81, 2.21)	6.6% (−4.5%, 18.9%)	0.259
Appliances and support and braces	33.0%	36.9%	0.78 (0.44, 1.25)	−5.5% (−16.3%, 5.4%)	0.321
Assistive devices	25.1%	25.2%	1.35 (0.81, 2.07)	6.1% (−3.7%, 15.9%)	0.222
Transcutaneous electric nerve stimulation (TENS)	16.2%	16.4%	0.98 (0.52, 1.82)	−0.3% (−7.1%, 9.9%)	0.944
Warmth, heat or cold application	61.2%	58.4%	1.10 (0.69, 1.75)	2.3% (−9.1%, 12.7%)	0.688

Results were derived through multiple imputation of missing data using chained equations with mixed models for estimating coefficients (hence, denominator population *n* = 525; except for # which included 390 participants classified as clinically overweight or obese (classified as having a BMI ≥ 25 kg m^−2^)).

Δ Absolute percent differences were calculated by applying odds ratios derived by logistic mixed regression adjusted for age, sex and practice size to percent figures for the reference (control group) (clustering by GP practice accounted for in the mixed model). % difference relates to % in intervention group – % in control group.

‡ Comprises written or verbal information about joint problem, information about treatments and advice on self-management of joint problem.

There was a reduction in self-reported use of oral NSAIDs in the intervention arm −15.6% (−28.3%, −3.5%), and less reliance on walking aids −13.9% (−24.6%, −1.6%), compared with the control arm.

The statistically non-significant findings for the primary clinical outcome measure (SF-12 PCS) were largely replicated in the three sensitivity analyses (see Supplementary file, Table I).

### Secondary outcomes

Differences between intervention and control were not statistically significant for most secondary outcomes (see Table IV). Evaluation of clinical markers of recovery (including responder criteria) showed no significant differences between groups (see Supplementary file, Table III). Of the significant differences in secondary outcomes, the Patient Enablement Score (mean (SD)) was greater in the intervention arm compared with the control arm at 6 months (3.21 (3.44) vs 2.29 (2.96)), and also at the secondary endpoints of three and 12 months. By contrast PASE scores indicated a fall in reporting of physical activity in the intervention arm compared with control (statistically significant for the walking domain at three and 6 months (data not shown)) but this was not clinically significant. In those participants receiving both the GP and practice nurse consultations, there was an increase in the use of strengthening exercises at 3 months (data not shown).

**Table IV:** Effectiveness of the model osteoarthritis consultation compared to usual primary care for osteoarthritis: evaluation of secondary outcomes

Outcome	Intervention			Control			∗Mean difference (95% CI)	†Effect size (95% CI)	*P*-value
	Valid *n*	Mean	SD	Valid *n*	Mean	SD			
**Pain intensity scores**									
**Hip**									
Post-consultation	274	3.52	3.47	234	3.38	3.34	–	–	–
3 months	241	2.98	3.16	190	3.04	3.28	−0.19 (−0.63, 0.26)	−0.06 (−0.18, 0.08)	0.415
6 months	212	2.59	3.09	172	2.78	3.17	−0.24 (−0.78, 0.30)	−0.07 (−0.23, 0.09)	0.382
12 months	187	2.79	3.13	155	2.71	2.97	−0.15 (−0.90, 0.59)	−0.04 (−0.26, 0.17)	0.687
**Knee**									
Post-consultation	278	5.67	3.09	230	5.63	3.28	–	–	–
3 months	247	4.64	3.11	195	4.69	3.14	−0.49 (−0.94, −0.05)	−0.15 (−0.30, −0.02)	0.031
6 months	215	4.27	3.01	173	4.68	3.17	−0.20 (−0.74, 0.34)	−0.06 (−0.23, 0.11)	0.468
12 months	190	4.25	3.32	159	3.89	3.08	0.04 (−0.71, 0.80)	0.01 (−0.22, 0.25)	0.909
**Hand**									
Post-consultation	273	2.94	3.11	230	2.99	3.22	–	–	–
3 months	245	2.62	2.83	194	2.61	2.86	−0.15 (−0.56, 0.25)	−0.05 (−0.18, 0.08)	0.458
6 months	213	2.57	2.78	170	2.89	3.02	−0.09 (−0.57, 0.38)	−0.03 (−0.18, 0.12)	0.697
12 months	189	2.80	2.86	157	2.91	3.02	−0.40 (−1.04, 0.24)	−0.13 (−0.33, 0.08)	0.218
**Foot**									
Post-consultation	275	2.79	3.12	231	2.97	3.34	–	–	–
3 months	245	2.36	2.85	192	2.44	3.04	0.03 (−0.41, 0.47)	0.01 (−0.13, 0.15)	0.904
6 months	209	2.43	2.92	170	2.46	3.08	0.30 (−0.23, 0.83)	0.09 (−0.07, 0.26)	0.272
12 months	189	2.48	3.05	157	2.45	3.14	0.27 (−0.45, 0.99)	0.08 (−0.14, 0.31)	0.461
**WOMAC physical function**									
Post-consultation	283	12.28	7.61	233	12.09	6.87	–	–	–
3 months	250	10.56	7.73	196	10.32	6.84	−0.36 (−1.24, 0.52)	−0.05 (−0.17, 0.07)	0.417
6 months	219	9.67	7.21	175	10.46	7.10	−0.53 (−1.68, 0.61)	−0.07 (−0.23, 0.08)	0.362
12 months	191	10.24	7.53	161	9.28	6.65	0.13 (−1.64, 1.90)	0.02 (−0.23, 0.26)	0.884
**AIMS 2 hand & finger function**									
Post-consultation	279	1.62	2.04	233	1.82	2.53	–	–	–
3 months	243	1.64	2.09	197	1.56	2.05	0.16 (−0.16, 0.48)	0.07 (−0.07, 0.21)	0.331
6 months	220	1.55	2.10	175	1.73	2.26	0.02 (−0.35, 0.39)	0.01 (−0.15, 0.17)	0.932
12 months	192	1.51	2.11	161	1.70	2.21	−0.17 (−0.67, 0.33)	−0.07 (−0.29, 0.14)	0.505
**IPAQ**									
Post-consultation	200	2745	3285	171	3125	3830	–	–	–
3 months	182	2378	2912	157	3306	4073	−693 (−1447, 60)	−0.20 (−0.41, 0.02)	0.071
6 months	181	2200	2967	144	2519	2787	−629 (−1397, 139)	−0.18 (−0.41,0.02)	0.108
12 months	167	2356	2414	142	3041	3460	−595 (−1396, 207)	−0.17 (−0.39,0.06)	0.146
**Physical activity for the elderly (PASE)**									
Post-consultation	237	138.7	75.9	195	147.5	85.3	–	–	–
3 months	203	123.6	72.0	176	149.1	90.6	−22.1 (−35.7, −8.5)	−0.28 (−0.44, −0.11)	0.001
6 months	190	123.0	68.7	143	136.2	73.2	−18.3 (−34.0, −2.6)	−0.23 (−0.42, −0.03)	0.022
12 months	157	134.2	69.6	142	148.2	77.9	−17.0 (−38.2, 4.1)	−0.21 (−0.48, 0.05)	0.127
**PHQ8**									
Post-consultation	286	5.02	5.24	235	4.45	4.65	–	–	–
3 months	248	4.36	4.50	199	3.85	4.64	0.38 (−0.29, 1.04)	0.08 (−0.06, 0.21)	0.265
6 months	223	4.07	4.87	174	4.29	4.74	0.02 (−0.74, 0.78)	0.00 (−0.15, 0.16)	0.965
12 months	194	4.06	4.74	162	3.96	4.81	−0.16 (−1.18, 0.86)	−0.03 (−0.24, 0.17)	0.759
**GAD7**									
Post-consultation	273	3.70	4.89	231	3.22	4.33	–	–	–
3 months	242	3.16	4.32	195	2.90	4.60	−0.07 (−0.72, 0.58)	−0.02 (−0.15, 0.12)	0.825
6 months	212	2.72	4.05	172	2.73	4.28	0.60 (−0.15, 1.35)	0.13 (−0.03, 0.29)	0.115
12 months	187	2.90	4.31	159	2.75	3.84	−0.45 (−1.47, 0.57)	−0.10 (−0.32, 0.12)	0.388
**SF-12 MCS**									
Post-consultation	280	50.24	11.34	231	51.14	10.91	–	–	–
3 months	250	51.04	10.74	204	50.91	11.13	0.09 (−1.64, 1.82)	0.01 (−0.15, 0.16)	0.917
6 months	229	50.90	10.81	180	50.79	10.66	−0.18 (−2.11, 1.75)	−0.02 (−0.19, 0.16)	0.853
12 months	200	51.49	10.74	166	51.34	10.11	0.08 (−2.39, 2.55)	0.01 (−0.22, 0.23)	0.947
**Arthritis self-efficacy pain subscale**									
Post-consultation	282	5.40	1.99	232	5.39	2.11	–	–	–
3 months	246	5.82	2.18	190	5.82	2.13	−0.13 (−0.50, 0.25)	−0.06 (−0.25, 0.12)	0.516
6 months	218	5.86	2.08	173	5.82	2.31	0.00 (−0.44, 0.43)	0.00 (−0.22, 0.21)	0.984
12 months	197	5.83	2.24	157	6.04	2.17	−0.15 (−0.74, 0.44)	−0.07 (−0.36, 0.22)	0.615
**Patient enablement**									
Post-consultation	–	–	–	–	–	–	–	–	–
3 months	253	2.82	3.16	202	2.61	3.25	0.86 (0.10, 1.63)	0.27 (0.03, 0.51)	0.027
6 months	224	3.21	3.44	178	2.29	2.96	1.34 (0.59, 2.10)	0.42 (0.18, 0.65)	<0.001
12 months	198	2.80	3.18	162	2.59	3.19	0.88 (0.05, 1.71)	0.27 (0.02, 0.53)	0.039

ICC (unadjusted): Hip pain <0.001; Knee pain <0.001; Hand pain 0.003; Foot pain 0.016; WOMAC-pf <0.001; AIMS <0.001; IPAQ <0.001; PASE <0.001; PHQ <0.001; GAD <0.001; SF-MCS 0.001; self-efficacy 0.001; patient enablement 0.010.

∗ Calculated as mean difference for Intervention – Control score by linear mixed modelling adjusted for age, gender, practice size and corresponding baseline measures (clustering by GP Practice accounted for in the mixed model).

† Mean difference relative to pooled ‘baseline’ (post-consultation) SD except for patient enablement for which the relative SD was that of the SD at follow up (since no baseline patient enablement was collected).

### Treatment fidelity

At 3 months, self-reported consultations with a practice nurse for joint problems had occurred in *n* = 70 (28.9%) in the intervention arm compared with *n* = 26 (13.5%) in the control arm.

### Adverse events

No adverse events were reported as a result of the interventions.

## Discussion

In this cluster-randomised controlled trial there was no evidence of benefit of this intervention, as delivered in this pragmatic randomised trial, on the primary clinical outcome (physical functioning) at 6 months after adjustment for predefined potential confounders. However, there were significant increases in uptake and use of NICE OA core recommendations in intervention practices compared with control over 6 months. Use of oral NSAIDs was reduced in participants in the intervention arm.

We developed three primary care innovations in preparation for this trial: the MOAC, training to deliver the consultation, and an e-template specifically for use during consultations with patients who have OA. The model consultation consisted of an OA guidebook^13^, ^20^; an enhanced OA consultation with a GP^15^; and subsequent follow up with a practice nurse in a dedicated OA clinic. The training for healthcare professionals was developed to implement delivery of the enhanced OA consultation^15^; and the e-template was developed to record quality measures of OA care^9^, ^21^. These three innovations provided the tools for implementing NICE Quality Standards for OA in general practice^22^.

Clinical guidelines represent a distillation of best evidence about either clinically effective interventions and management determined by expert consensus to represent best practice, such as information provision. The challenge for clinicians and policy makers is to get such guidelines adopted in practice. Our novel intervention has achieved substantial improvements in adoption of the guidelines in primary care, and in achieving markers of quality of care for patients with OA. Although there was a substantial increase in guideline uptake, there remains a need to achieve universally good adoption of recommended management options^9^.

Despite implementation successes in this trial, the expected improvement in clinical outcomes did not occur. There are a number of possible explanations. First, it is possible that the lack of effect on clinical outcomes reflects a genuine lack of intervention efficacy. Considering the WISE theoretical framework as applied to MOSAICS^23^, this could relate to the Guidebook, to the responsiveness of professionals, or to access to care (the nurse follow-up consultations). As the cost of providing nurse clinics was reimbursed, and in some cases staff were directly provided, it seems unlikely that insufficient clinic availability was the cause of low uptake but other service pressures or patient or clinician beliefs about OA may have affected access to the practice nurse^7^. The GP remained the gatekeeper for referral and this could be the main reason why more patients didn't see the nurse.

Most participants had multisite, chronic, joint pain so perhaps it was unrealistic to expect changes in a primary outcome with an endpoint of 6 months for a long-term condition, particularly if patients were not already engaged in positive lifestyle behaviours. More specialist clinical services and referral for specialist pain-management may have been indicated for some.

Secondly, the clinical outcome measures used may be inappropriate in routine practice for patients with multisite OA and multiple morbidities, who may be different to participants in OA clinical trials; in particular in the guidelines the evidence base may have been drawn from a narrower clinical spectrum of OA. Thirdly, the ‘dose’ of the intervention in practice may have been insufficient to improve long-term pain and disability – for example less than a third of participants reported consulting the nurse, the focus of the intervention was on supporting self-management, and uptake of exercise (known to be clinically effective for OA^24^) may not have been of sufficient intensity to achieve additional changes in the SF-12 PCS. Finally, closing the gap between uptake of guideline evidence and primary care practice may benefit from multiple strategies, and the best way of combining strategies is unknown^25^. Further work is still needed to explore how optimal OA management can be provided in primary care.

Of the secondary outcomes, improvement in patient enablement suggests a beneficial effect of the intervention on the capacity of patients for self-management – one of the targets of NICE core guidance.

### Strengths and limitations

Bias in cluster trials due to differential selection of patients between intervention and control arms is a recognised problem^26^. We designed our cluster trial to address these challenges by including a pre-recruitment population survey mailed to 30,000 community dwelling adults aged ≥45 years registered in primary care in order to identify potentially eligible participants prior to any consultation about OA. When any of the individuals subsequently consulted their GP with peripheral joint pain, and the GP entered a relevant Read code into the patient's electronic patient record, the patient automatically became eligible for the trial and was posted a baseline post-consultation questionnaire to complete.

By removing the process of eligibility checking and recruitment from the GP, we reduced the likelihood of selection bias between intervention and control practices. However, in the intervention practices in this pragmatic trial, it was clear that GPs had been selective to some extent in their referral of patients for practice nurse consultation, although numbers were too small to ascertain on which characteristics patients were selected. Other design strengths included randomisation procedures, blinding of the research nurse and use of minimal data in follow-up. Another strength of the MOSAICS study was its use of implementation theories^9^, ^15^, ^27^, ^28^, ^29^, ^30^.

Patient and Public Involvement and Engagement was used extensively throughout the MOSAICS study, including in co-application for funding, steering group membership, OA guidebook development and selection of measures of self-reported quality of OA primary care^9^, ^17^, ^31^.

A weakness of our study was that not all participants recruited in the intervention arm received the linked practice nurse consultation, which could have diluted the impact of the intervention. Less than a third of participants in the intervention arm saw the practice nurse; further analysis did not reveal a clear underlying reason for this.

Since the NICE 2008 guidelines^2^, upon which the MOSAICS trial was based, the evidence about the role of paracetamol in the pharmacological management of OA has been questioned. Paracetamol was promoted as a first-line pharmacological therapy along with topical NSAIDs, and remains a recommended option in the NICE 2014 guidelines. A recent systematic review^32^ concluded that paracetamol adds little to the management of OA and does have risks.

Jordan *et al.* previously described the practice-level evaluation of the intervention in the cluster trial using the anonymised medical records^10^. In practice records, supply of written information increased in the intervention practices but remained stable in the control practices^10^. We found similar results here in the patient-reported QIs. Comparisons can also be made with other studies of self-management in primary care. Kennedy *et al.*^14^, implementing the WISE model of self-management support in primary care, found a lack of clinical benefit. However, our intervention was more intense, and we were able to detect change in quality indicators of care – not measured by Kennedy *et al.*^14^ – by using patient self-report. These included an increase in non-pharmacological approaches and a decline in use of oral NSAIDs. Unlike the study of self-management by Buszewicz *et al.*^33^, which improved participants' perceived self-efficacy to manage symptoms, we did not find a benefit for pain self-efficacy. However, we did notice an improvement in patient enablement scores which could be regarded consistent with Buszewicz's observation. A German cluster trial of self-management support by GPs in adults aged ≥18 years with hip and knee OA noted improvements in quality of life associated with the addition of a practice nurse telephone follow-up to support self-management^34^. Further models of implementing OA guidelines have been described, compared and contrasted^35^.

In conclusion, although our novel method of delivering and supporting self-management for OA in general practice increased the uptake of quality standards of OA care, there was no evidence of benefit of this intervention on the primary outcome of physical functioning at 6 months.

## Declarations

### Ethics approval and consent to participate

The study was approved by the North West 1 Research Ethics Committee, Cheshire (REC reference: 10/H1017/76) and monitored by an Independent Trial Steering Committee and Data Monitoring Committee (Trial registration number ISRCTN06984617). Trial registration status on the Register is ‘retrospective’ but recruitment of the first patient into the cluster RCT is clearly recorded on the Register as occurring on 11th May 2012, a date after the registration date of July 2011 (see Registry entry update 11/07/2016).

## Consent for publication

Not applicable.

### Availability of data and materials

The Centre has established data sharing arrangements to support joint publications and other research collaborations. Applications for access to anonymised data from our research databases are reviewed by the Centre's Data Custodian and Academic Proposal (DCAP) Committee and a decision regarding access to the data is made subject to the NRES ethical approval first provided for the study and to new analysis being proposed. Further information on our data sharing procedures can be found on the Centre's website (http://www.keele.ac.uk/pchs/publications/datasharingresources/) or by emailing the Centre's data manager (primarycare.datasharing@keele.ac.uk).

## Authors' contributions

KD was the Principal Investigator of the study, designed the study, oversaw the conduct and delivery of the study, and drafted the manuscript. All authors were involved in design and delivery of the study, and revisions of the paper. EKA wrote the analysis plan, cleaned the data, and carried out the analysis with senior support from ML; MP and EH led development of the intervention; GM, SR, AF, AM and ZP contributed to delivery of training; JJE designed the electronic template; CJ reported PPI involvement; APR coordinated the study; EMH was Chief Investigator for the NIHR programme within which this study was nested. All authors have approved the final version.

## Conflict of interest

The study funding is detailed below. KD was a member of the NICE Osteoarthritis Guidelines Development Group CG 59 (2008) and CG 177 (2014) (with MP), and a member of the NICE Quality Standards Group for Osteoarthritis. KD has been an invited speaker at Bone and Joint Decade 2015 Conference in Oslo and Osteoarthritis Research Society International. KD also received a grant from EIT-Health for implementation.

The lead author (Dziedzic) affirms that this manuscript is an honest, accurate and transparent account of the study being reported; that no important aspects of the study have been omitted; and that any discrepancies from the study as planned have been explained.

## Funding

This paper presents independent research funded by the National Institute for Health Research (NIHR) Programme Grant (RP-PG-0407-10386).

The views expressed in this paper are those of the author(s) and not necessarily those of the NHS, the NIHR or the Department of Health. This research was also funded by the Arthritis Research UK Centre in Primary Care grant (Grant Number 18139). KD, ELH and CJ are part-funded by the National Institute for Health Research (NIHR) Collaborations for Leadership in Applied Health Research and Care West Midlands. KD is part-funded by a Knowledge Mobilisation Research Fellowship (KMRF- 2014-03-002) from the NIHR. AF was supported by an NIHR Doctoral, Clinical Academic Training Fellowship. JJE is a NIHR Academic Clinical Lecturer in Primary Care and was supported by the NIHR through an In-Practice Fellowship. EMH is a NIHR Senior Investigator.

## Role of the funding source

The study funder had no role in the design, collection of data, analysis, interpretation of data, writing of the manuscript or decision to publish.

## Patient and public involvement and engagement (PPIE)

The Arthritis Research UK Primary Care Centre at Keele is committed to taking an explicit and systematic approach to involving patients and the public in research^31^. For this trial, a Research User Group worked in collaboration with researchers on a wide range of tasks including: development and design of the OA guidebook^13^ developing training for GPs and practice nurses, grant co-applicant and Steering Committee Membership.

## Figures and Tables

**Table:** 

Participant eligibility criteria	
*Patients	*Inclusion criteria:* • Males and Females • 45 years and over • Registered with a MOSAICS study practice • Joint pain in the past year (in the hand, hip, knee, foot) self-reported in the Health Survey • Consent to further contact from the study team (consent sought as part of the Health Survey) • Consent to medical record review (consent sought as part of the Health Survey) • Consulting with joint pain in the cluster trial recruitment period • Triggered the e-template in EMIS system in the consultation • Returned post-consultation baseline Questionnaire *Exclusion criteria:* • Excluded via GP screen of practice list • Unable to give fully informed consent e.g., learning difficulties or dementia • Resident in a care or nursing home • History of serious disease e.g., malignancy, terminal illness • Unable to consult in the general practice surgery • Flagged as excluded from research in that practice • Declined to take part in the post-consultation baseline Questionnaire

* All patients registered with MOSAICS practices randomised to the intervention arm of the study had access to the GP and practice nurse clinics, including those who did not consent to be followed up, as these ran as part of normal clinical care within each intervention practice.

Key: EMIS = Egton Medical Information System; GP = General Practitioners; MOSAICS = Management of Osteoarthritis In Consultations Study.

## Appendix 2 The model OA consultation (MOAC) intervention and training

The MOAC provided a new service for patients in general practice to enhance the management of OA based on the NICE OA guidelines^2^, ^3^. The model consultation was a linked GP and practice nurse integrated consultation supported by the use of an OA Guidebook^13^. The aim of the MOAC was to operationalise in general practice three aspects of care for OA:

1. The three core treatments of the NICE OA Guideline (2008)^2^: verbal and written information, advice to exercise and increase physical activity, interventions to achieve weight loss.
2. The NICE OA Guideline recommendations for first-line analgesia: paracetamol and topical NSAIDs.
3. Support for self-management of OA based upon the Whole Systems Informing Self-Management Engagement (WISE) model^14^, which centres on the provision of knowledge for patients and a style of intervention built on professional responsiveness to patients' needs.

### Model OA consultation with the GP

Patients aged 45 years and over with peripheral joint pain (knee, hip, hand, and foot) had an initial consultation with the GP. An OA e-template was triggered as part of the consultation and GPs were asked to assess and make a clinical diagnosis of the problem without the routine use of X-ray. GPs were then asked to offer an explanation of OA (in suitable language and tailored to the patient's level of understanding and individual circumstances) and offer first line analgesia as appropriate (paracetamol; topical NSAIDS). An OA guidebook (http://www.keele.ac.uk/media/keeleuniversity/ri/primarycare/pdfs/OA_Guidebook.pdf) written by patients and health care professionals for patients was given to the patient. It offers support for self-management, promotes the NICE core treatments and provides accounts of how people live with OA. The GP was then asked to explain the next steps: for the patient to read the OA guidebook and to arrange a follow-up appointment with the practice nurse.

### Model OA consultation with the practice nurse (nurse-led OA clinic)

The timing of the first appointment with the practice nurse was planned for a minimum of 2 weeks after the initial GP consultation. This gave patients time to read the guidebook and try those self-management strategies they felt were suitable. In the first consultation the practice nurse was asked to refer to the guidebook as a resource to answer questions and clarify issues, ascertain the advice from the GP consultation, negotiate and agree appropriate goals, discuss the need for pain relief and opportunities for healthy eating, physical activity and exercise as appropriate.

The timing of up to three follow-up visits with the nurse was agreed between the patient and the practice nurse, but was scheduled to be delivered within 3 months following the GP consultation. The follow-up practice nurse consultations were tailored to the patient's individual needs and could focus on, for example, reviewing the self-management plan, demonstrating exercises (Arthritis Research UK Exercises for Arthritis leaflet), giving advice as to how this could be maintained longer-term or making any necessary referrals to the broader multidisciplinary team. The practice nurse consultations were supported by a specifically tailored Case Report Form (available on request) and a nurse toolkit that included advice leaflets to give to patients (content of the toolkit available on request).

### Training

Training and educational packages were developed for GPs and practice nurses by drawing on the work of May *et al.*^28^, Grol^29^ and Michie *et al.*^30^ Intervention practices received practice updates on core NICE recommendations for OA (diagnosis; written information [an OA guidebook], exercise and physical activity, healthy eating, pain management). GPs received training on the delivery of the initial consultation for a new or established patient during four practice-based sessions (2 h ×3, 1 h ×1) utilising simulated patients in skills training sessions^15^. Practice nurses received 4 days of training on how to support patients to self-manage OA, using a patient-centred approach, the OA guidebook, goal setting, pain management (analgesia and exercise) and the core NICE recommendations (information and advice, strengthening exercise and aerobic fitness training, and weight management), again with the use of simulated patients.

Members of the wider multidisciplinary team linked to the intervention practices (e.g., physiotherapists, occupational therapists, podiatrists, pharmacists) were invited to a workshop to inform them of the use of the MOAC intervention in their local general practice and its aims. They were given the OA guidebook as reference material, however no change to usual care was instigated.

## Appendix A Supplementary data

mmc1 mmc1

## References

[1] Global Burden of Disease Study 2013 Collaborators (2015 Aug 22). Global, regional, and national incidence, prevalence, and years lived with disability for 301 acute and chronic diseases and injuries in 188 countries, 1990–2013: a systematic analysis for the Global Burden of Disease Study 2013. Lancet.

[2] National Institute for Health & Clinical Excellence (2008). NICE Clinical Guideline [CG59] Osteoarthritis: The Care and Management of Osteoarthritis in Adults.

[3] National Institute for Health & Care Excellence (2014). NICE Clinical Guideline [CG177] Osteoarthritis: Care and Management in Adults.

[4] Steel N., Bachmann M., Maisey S., Shekelle P., Breeze E., Marmot M. (2008). Self reported receipt of care consistent with 32 quality indicators: national population survey of adults aged 50 or more in England. BMJ.

[5] McHugh G.A., Silman A.J., Luker K.A. (2007). Quality of care for people with osteoarthritis: a qualitative study. J Clin Nurs.

[6] Mann C., Gooberman-Hill R. (2011). Health care provision for osteoarthritis: concordance between what patients would like and what health professionals think they should have. Arthritis Care Res (Hoboken).

[7] Paskins Z., Sanders T., Croft P.R., Hassell A.B. (2015). The identity crisis of osteoarthritis in general practice: a qualitative study using video-stimulated recall. Ann Fam Med.

[8] Papandony M.C., Chou L., Seneviwickrama M., Cicuttini F.M., Lasserre K., Teichtahl A.J. (2017 Jul). Patients' perceived health service needs for osteoarthritis (OA) care: a scoping systematic review. Osteoarthritis Cartilage.

[9] Dziedzic K.S., Healey E.L., Porcheret M., Ong B.N., Main C.J., Jordan K.P. (2014). Implementing the NICE osteoarthritis guidelines: a mixed methods study and cluster randomised trial of a model osteoarthritis consultation in primary care – the Management of OsteoArthritis in Consultations (MOSAICS) study protocol. Implement Sci.

[10] Jordan K.P., Edwards J.J., Porcheret M., Healey E.L., Jinks C., Bedson J. (2017). Effect of a model consultation informed by guidelines on recorded quality of care of osteoarthritis (MOSAICS): a cluster randomised controlled trial in primary care. Osteoarthritis Cartilage.

[11] Edwards J.J., Jordan K.P., Peat G., Bedson J., Croft P.R., Hay E.M. (2015). Quality of care for OA: the effect of a point-of-care consultation recording template. Rheumatology (Oxford).

[12] Porcheret M., Grime J., Main C., Dziedzic K. (2013). Developing a model osteoarthritis consultation: a Delphi consensus exercise. BMC Musculoskelet Disord.

[13] Grime J., Dudley B. (2014). Developing written information on osteoarthritis for patients: facilitating user involvement by exposure to qualitative research. Health Expect.

[14] Kennedy A., Bower P., Reeves D., Blakeman T., Bowen R., Chew-Graham C. (2013). Implementation of self management support for long term conditions in routine primary care settings: cluster randomised controlled trial. BMJ.

[15] Porcheret M., Main C., Croft P., McKinley R., Hassell A., Dziedzic K. (2014). Development of a behaviour change intervention: a case study on the practical application of theory. Implement Sci.

[16] Ware J., Kosinski M., Keller S.D. (1996). A 12-Item Short-Form Health Survey: construction of scales and preliminary tests of reliability and validity. Med Care.

[17] Blackburn S., Higginbottom A., Taylor R., Bird J., Østerås N., Hagen K.B. (2016). Patient-reported quality indicators for osteoarthritis: a patient and public generated self-report measure for primary care. Res Involv Engagem.

[18] Howie J.G.R., Heaney D.J., Maxwell M., Walker J.J. (1998). A comparison of a Patient Enablement Instrument (PEI) against two established satisfaction scales as an outcome measure of primary care consultations. Fam Pract.

[19] Pham T., Van Der Heijde D., Lassere M., Altman R.D., Anderson J.J., Bellamy N. (2003). Outcome variables for osteoarthritis clinical trials: the OMERACT/OARSI set of responder criteria. J Rheumatol.

[20] Morden A., Jinks C., Ong B.N., Porcheret M., Dziedzic K.S. (2014 Dec 13). Acceptability of a ‘guidebook’ for the management of Osteoarthritis: a qualitative study of patient and clinician's perspectives. BMC Musculoskelet Disord.

[21] Edwards J.J., Khanna M., Jordan K.P., Jordan J.L., Bedson J., Dziedzic K.S. (2015). Quality indicators for the primary care of osteoarthritis: a systematic review. Ann Rheum Dis.

[22] National Institute for Health & Care Excellence (2015). Quality Standard for Osteoarthritis (NICE Quality Standard 87).

[23] Dziedzic K.S., French S., Davis A.M., Geelhoed E., Porcheret M. (2016). Implementation of musculoskeletal Models of Care in primary care settings: theory, practice, evaluation and outcomes for musculoskeletal health in high-income economies. Best Pract Res Clin Rheumatol.

[24] Uthman O.A., van der Windt D.A., Jordan J.L., Dziedzic K.S., Healey E.L., Peat G.M. (2013). Exercise for lower limb osteoarthritis: systematic review incorporating trial sequential analysis and network meta-analysis. BMJ.

[25] Lau R., Stevenson F., Ong B.N., Dziedzic K., Treweek S., Eldridge S. (2015). Achieving change in primary care–effectiveness of strategies for improving implementation of complex interventions: systematic review of reviews. BMJ Open.

[26] Eldridge S., Kerry S. (2012).

[27] Nilsen P. (2015). Making sense of implementation theories, models and frameworks. Implement Sci.

[28] May C., Finch T., Mair F., Ballini L., Dowrick C., Eccles M. (2007). Understanding the implementation of complex interventions in health care: the normalization process model. BMC Health Serv Res.

[29] Grol R. (1997). Personal paper. Beliefs and evidence in changing clinical practice. BMJ.

[30] Michie S., Johnston M., Francis J., Hardeman W., Eccles M. (2008). From theory to intervention: mapping theoretically derived behavioural determinants to behaviour change techniques. Appl Psychol Int Rev Appliquee-Revue Int.

[31] Jinks C., Higginbottom A., Rhodes C., Ong P., Dziedzic K.S. (2014). The MOSAICS study. NIHR School for Primary Care Research Ed. Patient and Public Involvement: Case Studies in Primary Care Research.

[32] Roberts E., Delgado Nunes V., Buckner S., Latchem S., Constanti M., Miller P. (2016). Paracetamol: not as safe as we thought? A systematic literature review of observational studies. Ann Rheum Dis.

[33] Buszewicz M., Rait G., Griffin M., Nazareth I., Patel A., Atkinson A. (2006). Self management of arthritis in primary care: randomised controlled trial. BMJ.

[34] Rosemann T., Joos S., Laux G., Gensichen J., Szecsenyi J. (2007). Case management of arthritis patients in primary care: a cluster-randomized controlled trial. Arthritis Rheum.

[35] Allen K.D., Choong P.F., Davis A.M., Dowsey M.M., Dziedzic K.S., Emery C. (2016). Osteoarthritis: models for appropriate care across the disease continuum. Best Pract Res Clin Rheumatol.

