# Supplementary material for: Implementing core NICE guidelines for osteoarthritis in primary care with a model consultation (MOSAICS): a cluster randomised controlled trial
Source: Osteoarthritis Cartilage. 2018 Jan;26(1):43–53. doi: 10.1016/j.joca.2017.09.010 (PMC5759997; doi:10.1016/j.joca.2017.09.010)
Supplement: mmc1 [file mmc1.docx]

**Supplementary Tables S1, S2, & S3**

**Supplementary Table S1: Effectiveness of the Model Osteoarthritis Consultation compared to usual primary care for osteoarthritis: Sensitivity evaluation of primary outcome measure (SF-12 PCS) at 6 months**

|  | **Mean**  **difference** | **95% CI** | **P-value** |
| --- | --- | --- | --- |
| * Mixed-model with level 3 (highest level) General Practices replaced by GP practitioners | -0.39 | -2.31, 1.54 | 0.696 |
| † **CACE** | -0.11 | -4.96, 4.74 | 0.963 |
| **‡** Two-stage cluster level analysis | n/a | 0.824, 0.838 | 0.832 |

Sensitivity analyses were conducted for the primary clinical outcome (SF-12 PCS). First, as the number of clusters was small, general practice as cluster-variable was replaced by individual GPs as a random factor in the analysis. Second, a Complier Average Causal Effect (CACE) analysis using a two-stage regression approach was performed to provide an unbiased treatment effect estimate for participants treated as per protocol specification (treatment administered as per protocol in the intervention arm was based on participants having seen the practice nurse in the intervention practices). Lastly, a cluster-level analysis was undertaken using a permutation test.

* Calculated as mean difference for Intervention group - control group by linear mixed modelling adjusted for age, gender, practice size and baseline SF12-PCS (clustering by GP Practitioners accounted for in the mixed model)

† CACE – Complier Average Causal Effect

‡ Cluster level analysis using a non-parametric permutation test. n/a not applicable

**Supplementary Table S2: Effectiveness of the Model Osteoarthritis Consultation compared to usual primary care for osteoarthritis: Sub-group evaluation of** **arthritis self-efficacy scale**

|  | **Intervention** | | **Control** | |  |  |  |
| --- | --- | --- | --- | --- | --- | --- | --- |
| **Arthritis self-efficacy** | **Age group (>=65 vs. <65 years)** | | | | | | |
|  | **<65**  Mean (SD), n | **>=65**  Mean (SD), n | **<65**  Mean (SD), n | **>=65**  Mean (SD), n | **Mean* difference** | **95% CI** | **P-value** |
| 3 months | 6.04 (2.32), 105 | 5.66 (2.05), 141 | 6.07 (2.19), 72 | 5.67 (2.09), 118 | 0.31 | -0.48, 1.09 | 0.443 |
| 6 months | 6.39 (1.99), 98 | 5.43 (2.05), 120 | 5.92 (2.30), 63 | 5.77 (2.31), 110 | -0.51 | -1.35, 0.33 | 0.238 |
| 12 months | 6.26 (2.32), 86 | 5.50 (2.12), 111 | 6.23 (2.37), 59 | 5.92 (2.04), 98 | -0.26 | -1.18, 0.66 | 0.578 |
| **Gender (female vs. male)** | | | | | | | |
|  | **Female**  Mean (SD), n | **Male**  Mean (SD), n | **Female**  Mean (SD), n | **Male**  Mean (SD), n | **Mean* difference** | **95% CI** | **P-value** |
| 3 months | 5.87 (2.12) 136 | 5.76 (2.27), 110 | 5.80 (2.13), 115 | 5.84 (2.14), 75 | 0.15 | -0.61, 0.91 | 0.697 |
| 6 months | 5.85 (2.07), 123 | 5.87 (2.09), 95 | 5.90 (2.37), 104 | 5.71 (2.21), 69 | 0.05 | -0.77, 0.87 | 0.911 |
| 12 months | 5.80 (2.27), 111 | 5.87 (2.19), 86 | 6.03 (2.19), 94 | 6.06 (2.15), 63 | -0.17 | -1.07, 0.73 | 0.712 |
| **Multi-site pain (1 vs. 2 or more sites)** | | | | | | | |
|  | **1 site**  Mean (SD), n | **2 or more sites**  Mean (SD), n | **1 site**  Mean (SD), n | **2 or more sites**  Mean (SD), n | **Mean* difference** | **95% CI** | **P-value** |
| 3 months | 6.79 (2.25), 67 | 5.54 (2.02), 172 | 6.61 (2.44), 50 | 5.53 (1.96), 135 | -0.46 | -1.43, 0.51 | 0.351 |
| 6 months | 6.64 (1.99), 67 | 5.52 (2.03), 145 | 7.34 (1.97), 42 | 5.30 (2.17), 127 | 0.59 | -0.43, 1.62 | 0.258 |
| 12 months | 6.83 (2.10), 58 | 5.44 (2.14), 134 | 7.28 (2.30), 50 | 5.46 (1.84), 107 | 0.30 | -0.79, 1.37 | 0.598 |
| **SF12-PCS median cut-off at baseline (<36.90 vs. >=36.90)** | | | | | | | |
|  | **<36.90**  Mean (SD), n | **>=36.90**  Mean (SD), n | **<36.90**  Mean (SD), n | **>=36.90**  Mean (SD), n | **Mean* difference** | **95% CI** | **P-value** |
| 3 months | 5.05 (2.01), 122 | 6.60 (2.03), 119 | 4.90 (1.80), 91 | 6.64 (2.06), 96 | -0.41 | -1.18, 0.37 | 0.302 |
| 6 months | 5.18 (2.00), 107 | 6.59 (1.89), 106 | 4.91 (2.15), 83 | 6.66 (2.13), 87 | -0.23 | -1.06, 0.61 | 0.594 |
| 12 months | 5.06 (2.20), 98 | 6.67 (1.95), 92 | 5.30 (2.02), 74 | 6.69 (2.10), 80 | -0.61 | -1.53, 0.30 | 0.189 |

* Calculated as mean difference for Intervention - Control score by linear mixed modelling adjusted for age, gender, practice size and baseline arthritis self-efficacy scale scores (clustering by GP Practices accounted for in the mixed model)

**Supplementary Table S3: Effectiveness of the Model Osteoarthritis Consultation compared to usual primary care for osteoarthritis: Evaluation of Global Change and Responder criteria**

|  | **Intervention** | | | **Control** | | | **3 months** | **6 months** | **12 months** |
| --- | --- | --- | --- | --- | --- | --- | --- | --- | --- |
|  | **3 months** | **6 months** | **12 months** | **3 months** | **6 months** | **12 months** | **OR**  **(95% CI)** | **OR**  **(95% CI)** | **OR**  **(95% CI)** |
| Global change* | 90 (35.5%) | 86 (36.7%) | 74 (35.7%) | 59 (28.5%) | 52 (29.8%) | 51 (29.8%) | 1.27 (0.70, 2.33)  p=0.432 | 1.13 (0.51, 2.52)  p=0.764 | 1.02 (0.44, 2.34)  p=0.967 |
| OMERACT/OARSI Responder‡ | 71 (27.6%) | 77 (34.7%) | 58 (31.7%) | 57 (28.4%) | 56 (30.3%) | 48 (32.0%) | 1.16 (0.69, 1.94)  p=0.566 | 1.26 (0.73, 2.16)  p=0.409 | 0.95 (0.52, 1.75)  p=0.879 |

* Global assessment of change dichotomised as: ‘completely recovered’/’much better’/’better’ relative to ‘no change’/’worse’/’much worse’ (reference category).

‡ OMERACT/OARSI responder criteria [19] combines pain intensity (0-10 scale average of 4 pain sites) and WOMAC function subscales and global assessment of change to determine if participants were ‘responders’ to treatment.

Pain intensity score ranges from 0 = no pain to 10 = pain as bad as could be, WOMAC function subscale score ranges from 0 = no difficulty to 32 = extreme degree of difficulty and global assessment of change was a 6 response categories item, and response ranges from 1 = completely recovered to 6 = much worse.

The criteria that qualify participants as ‘responders’ to treatment were as follows:

a) High improvement (relative change) in pain or in WOMAC function >=50% and absolute change >=20

or

b) Meeting at least 2 of the 3 following criteria:

- Improvement (relative change) in pain >=20% and absolute change >=10
- Improvement (relative change) in WOMAC function >=20% and absolute change >=10
- Participants reported they were better, much better, or completely recovered on global assessment of change item

## Absolute change in pain and WOMAC function were calculated as (pain at baseline - pain at follow-up), (WOMAC function at baseline - WOMAC function at follow-up) respectively.

## Relative change in pain and WOMAC function were calculated as (pain at baseline - pain at follow-up)/pain at baseline, (function at baseline - function at follow-up)/function at baseline respectively. Relative change were calculated after pain and WOMAC function measures were scaled from 1 to 101 to avoid dividing by 0 when calculating relative change.

Odds ratios calculated by logistic mixed-modelling adjusted for age, gender, practice size and baseline arthritis self-efficacy scale scores (clustering by GP Practices accounted for in the mixed model)
